# Inverted and Programmable Poynting Effects in Metamaterials

**DOI:** 10.1002/advs.202102279

**Published:** 2021-08-17

**Authors:** Aref Ghorbani, David Dykstra, Corentin Coulais, Daniel Bonn, Erik van der Linden, Mehdi Habibi

**Affiliations:** ^1^ Laboratory of Physics and Physical Chemistry of Foods Wageningen University Wageningen 6708WG The Netherlands; ^2^ Institute of Physics University of Amsterdam Amsterdam 1098XH The Netherlands

**Keywords:** mechanical metamaterials, normal and shear moduli, normal force, Poynting effect, torsion

## Abstract

The Poynting effect generically manifests itself as the extension of the material in the direction perpendicular to an applied shear deformation (torsion) and is a material parameter hard to design. Unlike isotropic solids, in designed structures, peculiar couplings between shear and normal deformations can be achieved and exploited for practical applications. Here, a metamaterial is engineered that can be programmed to contract or extend under torsion and undergo nonlinear twist under compression. First, it is shown that the system exhibits a novel type of inverted Poynting effect, where axial compression induces a nonlinear torsion. Then the Poynting modulus of the structure is programmed from initial negative values to zero and positive values via a pre‐compression applied prior to torsion. The work opens avenues for programming nonlinear elastic moduli of materials and tuning the couplings between shear and normal responses by rational design. Obtaining inverted and programmable Poynting effects in metamaterials inspires diverse applications from designing machine materials, soft robots, and actuators to engineering biological tissues, implants, and prosthetic devices functioning under compression and torsion.

## Graphical abstract

A rationally designed meta‐cylinder exhibits inverted and programmable Poynting effects. It shows nonlinear torsions under compression (inverted Poynting) and contractions under torsion. By applying a level of pre‐compression the structure can be programmed to induce tunable contraction or dilation (negative or positive Poynting effect) when twisted.

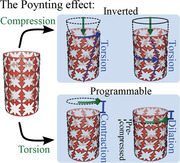

## 1 Introduction

**Figure 1:**
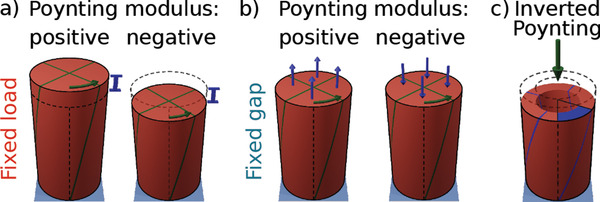
Poynting effect. a) Applying torsion under fixed loads causes dilation in a material with a positive Poynting modulus (left) and contraction in a material with a negative Poynting modulus (right). b) When the material is confined under a fixed gap, dilation and contraction will be manifested as positive (left) or negative (right) normal forces, respectively. c) Schematic of a cylindrical shell showing the inverted Poynting effect, where, in contrast to the Poynting effect's manifestation in Poynting's original experiment, an applied compression induces a nonlinear torsion in the cylinder.

The Poynting effect is a surprising non‐linear elastic effect that makes, in the original experiment of Poynting,^[^ ^1^ ^]^ a hanging piano wire under tension become longer when it is twisted (**Figure** **1**a, left). The consequence is also that if the distance between the two ends is fixed, the torsion induces a stress normal to the shear plane (normal stresses) that tends to separate the two ends (Figure 1b, left).^[^ ^2^, ^3^ ^]^ Developing normal stresses or axial deformations under torsion are two equivalent manifestations of the Poynting effect. Poynting found that the normal stress as a function of shear strain follows a quadratic relation with a positive coefficient,^[^ ^1^, ^4^, ^5^ ^]^ now called the Poynting modulus. While conventional materials such as the piano wires of Poynting show a positive Poynting modulus (Figure 1a,b, left), complex materials such as biopolymer systems^[^ ^6^, ^7^, ^8^, ^9^, ^10^, ^11^ ^]^ and designed structures^[^ ^12^ ^]^ can exhibit a negative Poynting modulus, causing a shear‐induced contraction under a fixed load (Figure 1a, right), or negative normal force at a fixed gap (Figure 1b, right and Video S1, Supporting Information). Designing metamaterials with exceptional mechanical properties originated from their structure rather than their composition has attracted much research in different disciplines of science.^[^ ^13^, ^14^ ^]^ So far, mechanical metamaterials have been studied mostly under compression or tension. The response of metamaterials to direct shear^[^ ^15^ ^]^ and in particular, the Poynting effect have remained largely unexplored. Understanding the complex coupling between shear and normal responses in metamaterials provides insights for harnessing and programming their shear and Poynting moduli.

Pulling or pushing on isotropic linear elastic objects causes expansion or contraction, but torsion is not allowed.^[^ ^16^, ^17^ ^]^ In a limited number of recent studies, induced linear torsion by compression have been uncovered in designed chiral structures^[^ ^18^, ^19^, ^20^, ^21^, ^22^ ^]^ mainly due to broken spatial symmetry of the system. Hence we ask whether an object is capable of transforming pure compression into a nonlinear torsion, which we call the inverted Poynting effect (Figure 1c and Video S1, Supporting Information). The inverted Poynting effect is not a conventional material property and it has not been observed so far.

In this work, we aim to program the sign and magnitude of the Poynting modulus in a designed structure. We design a cylindrical metamaterial that exhibits a programmable Poynting modulus and an inverted Poynting effect, where compression induces a torsion. The cylindrical metamaterial is composed of identical units (unit‐cells) consisting of beams with a suitably designed cross‐section profile. By applying compression, the cylinder induces nonlinear and linear torsions. The sign and magnitude of the normal and shear responses of the designed structure are tuned by the interplay between buckling instability and self‐contact interactions of the beams via a pre‐compression applied prior to shear deformation. We present a simple spring model that reproduces our experimental results and characterizes the essential design parameters to obtain the inverted Poynting and to program the Poynting modulus. Our findings outline a strategy towards the rational designing of a programmable nonlinear elastic response of metamaterials with potential applications in engineering biomaterials functioning under torsional deformation and designing robot arms, soft rotational actuators and mechanical switches.^[^ ^23^, ^24^, ^25^ ^]^ In soft robotics, for instance, coupling between torsion and compression can be exploited for the design of twisters, rotational actuators, kinematic controllers, and pick and place end‐effector.^[^ ^22^, ^26^ ^]^

## 2 System and Procedure

**Figure 2:**
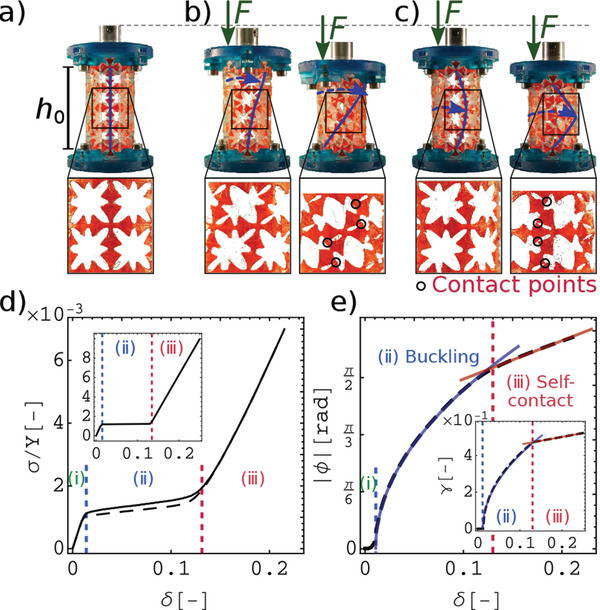
Experimental setup and compression tests. a) 3D printed structure is clamped between two plates. Unit‐cells and nonuniform beams are magnified. b,c) The compressed cylinder at the buckling (left) and self‐contact (right) regimes in torsion‐free (b) and clamped (c) compression experiments. Deformed unit‐cells are magnified. d) Rescaled nominal normal stress, σ/*Y*, as a function of compression strain, δ, for torsion‐free (dashed curve) and clamped (solid curve) experiments. e) Torsional angle, ϕ, as a function of compression with square root (blue) and linear (red) fits, in the buckling and self‐contact regimes, respectively. Insets are the predictions of the model and have the same axes and units as the main plots, except for the vertical axis in the inset of (e), which represents the shear strain in the 2D model, γ. γ can be converted to the equivalent torsion in our cylindrical shell via ϕ = (*h*_0_/*R*)γ. Vertical dashed blue and red lines show the onset of buckling and self‐contact regimes, respectively. Three regimes are marked with (i), (ii) and (iii).

We design a hollow cylindrical shell composed of an array of unit‐cells, which provides a network of nonuniform beams (**Figure**  **2**a) capable of side‐buckling and self‐contacting under compression.^[^ ^27^ ^]^ We apply compression and torsion deformations on the cylindrical metamaterial by clamping the structure between two custom‐made plates of an Anton Paar rheometer and measure the mechanical responses. The experimental details are explained in the experimental procedure section.

We first conduct two series of compression experiments with different boundary conditions: in the first series the bottom side of the shell is clamped and the top side is free to rotate (“torsion‐free”), while in the second series rotation is not allowed at both sides (“clamped”). Then we use the clamped boundary condition and perform two series of torsion experiments under fixed loads and fixed gaps.

The compression strain is defined as δ = |*h* − *h*_0_|/*h*_0_, where *h*_0_ = 40.1 mm is the initial effective height of the cylindrical shell and *h* is its height after the pre‐compression, under the compression force, *F*, with ±0.2% experimental error. Compression stress is defined by σ = *F*/*A*_*s*_, where *A*_*s*_ is the cross‐section area. Torsional angle, ϕ, develops by applying shear force, *F*_*s*_, and induces the axial deformation of δ_*n*_ under a fixed load or the normal force of *F*_*n*_ under a fixed gap. In isotropic elastic materials, the shear stress, σ_*s*_, is proportional to the shear strain, γ, σ_*s*_ = *G*_*s*_γ, and the normal stress induced by shear follows a quadratic relation as a function of shear strain, σ_*n*_ = *G*_*n*_γ^2^.^[^ ^1^, ^5^ ^]^ Here, we characterize the normal and shear responses of the structure with a Poynting modulus, *G*_*n*_, and a shear modulus, *G*_*s*_, respectively. Normal and shear force responses of a cylindrical shell under torsion are given by *F*_*n*_ = *G*_*n*_ *J*(ϕ/*h*)^2^, and *F*_*s*_ = τ/*R* = *G*_*s*_ *J*ϕ/(*Rh*), respectively, where τ is the torque around the axis of the shell, *R* = (*R*_*max*_ + *R*_*min*_)/2 is the mean radius, and $J=\frac{\pi }{2}\left(R_{max}^{4}-R_{min}^{4}\right)$ is the second moment of area of the shell.

## 3 Inverted Poynting Effect and Three Regimes of Structural Rearrangements

Our designed cylindrical metamaterial is capable of showing the inverted Poynting effect by inducing torsion under compression (Figure 2b,c). As shown in Figure 2b in the torsion‐free compression, we observe the rotational buckling with an affine torsional deformation across the height of the sample. However, in the clamped compression experiment, the torsional deformation accumulated at the middle of the structure (Figure 2c). In both compression experiments, three distinct regimes of configurational changes are observable in the structure: (i) pre‐buckling (Figure 2a), (ii) buckling of the beams (Figure 2b,c, left), and (iii) self‐contact (Figure 2b,c, right). Figure 2d, shows the compression stress, σ, rescaled by Young's modulus of the elastomer, Y = 3.63 MPa, as a function of compression strain, δ, for the torsion‐free (dashed curve) and clamped (solid curve) experiments, respectively. The three regimes have different effective stiffnesses due to their structural configurations. The effective stiffness of the system in each regime, *Y*_*eff*_, is determined by the slope of the curve times Young's modulus of the elastomer.

In the pre‐buckling regime (δ < δ_*b*_, where δ_*b*_ = 0.012 is the compression strain for the onset of the buckling regime) the vertical beams are stable and resist buckling (Figure 2a), resulting in a relatively high stiffness, with *Y*_*eff*_ = 0.095*Y*, in both compression tests. In the buckling regime (δ_*b*_ ⩽ δ < δ_*c*_, where δ_*c*_ = 0.13 is the compression strain for the onset of the self‐contact regime), the stress remains almost constant and the structure softens (*Y*_*eff*_ = 0.004*Y*), due to the buckling instability in the beams (Figure 2b,c, left). Finally, in the third regime (δ ⩾ δ_*c*_), the structure becomes stiff again, with *Y*_*eff*_ = 0.06, due to the self‐contact between the beams (Figure 2b,c, right).

In the torsion‐free experiments, compression induces shear deformation, with distinct behaviors at buckling and self‐contact regimes (Figure 2e and Video S2, Supporting Information). The coupling between the compression and torsion, in the buckling regime, is nonlinear and the best fit to the experimental torsional angle as a function of compression gives $\left|\phi \right|=4.9\sqrt{\delta -\delta_{b}}$ (blue curve in Figure 2e). The emergence of this square root relation is due to the buckling of the internal beams.^[^ ^28^ ^]^ However, in the self‐contact regime, compression and torsion are linearly proportional (red line in Figure 2e). Whereas linear coupling between compression and torsion has been achieved before for chiral structures,^[^ ^18^, ^19^, ^20^ ^]^ here for the first time nonlinear couplings between compression and torsion in an originally achiral structure have been studied. Thus, the cylindrical metamaterial translates an axial compression to a nonlinear torsion (the inverted Poynting effect) or linear torsion depending on the amount of compression. In the following, we investigate the Poynting response of the clamped structure under different loading conditions.

## 4 Poynting Modulus under Fixed Loads/Fixed Gaps

**Figure 3:**
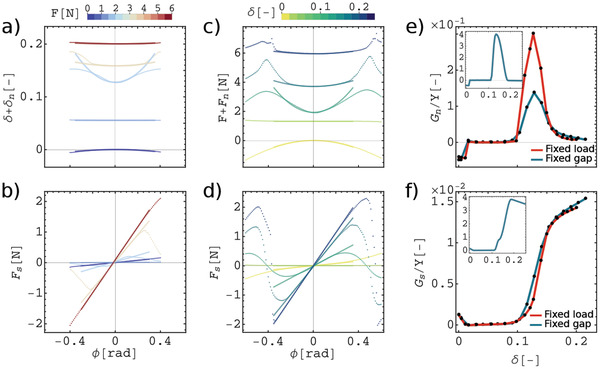
Poynting and shear moduli. a,b) Fixed‐load experiments: axial strain, δ + δ_*n*_, (a) and shear force, *F*_*s*_, (b) as a function of torsional angle, ϕ, while the structure is loaded under a fixed force (*F*, color scale). For the sake of clarity, only data for five experiments are shown. c,d) Fixed‐gap experiment: compression plus normal forces, *F* + *F*_*n*_, (c) and shear force, *F*_*s*_, (d) as a function of ϕ at different levels of pre‐compression strain (δ, color scale). e,f) Poynting (e) and shear (f) moduli rescaled by Young's modulus of the bulk, *Y*, as a function of pre‐compression strain, δ, for both loading scenarios calculated by fitting (solid curves) data points in a‐d. Insets are the modeling results in the fixed gap boundary condition and have the same axes and units as the main plots.

To quantify both manifestations of the Poynting effect for our metamaterial, we apply torsional deformation under fixed loads (Figure 1a) or fixed gaps (Figure 1b) and follow its normal responses. The first loading scenario is equivalent to Poynting's original experiment.^[^ ^1^ ^]^ Initially, the clamped structure is loaded under a force *F*, resulting in an axial compression strain, δ. Then the torsion is applied on the top boundary while *F* remains constant, causing an axial strain of δ_*n*_. The axial strain and applied shear forces are shown as a function of torsion for a range of loads in **Figure**  **3**a and b, respectively. To quantify the second manifestation of the Poynting effect (torsion under fixed gap), we apply torsion on the pre‐compressed shell while the height of the structure is fixed at *h*. Here, *F* is the force needed for the pre‐compression and *F*_*n*_ is the torsion‐induced normal force; *F* + *F*_*n*_ is the total axial force response of the pre‐compressed shell under torsion. Figure 3c,d shows the normal forces and applied shear forces as a function of torsion under fixed gaps, respectively (see Video S3, Supporting Information). For both series, the normal responses behave quadratically (Figure 3a,c) while the shear responses behave linearly (Figure 3b,d) as a function of torsion in the limit of small torsional deformation, ϕ <0.2 rad. Initially, the non‐compressed cylinder shows a contraction/negative normal force under torsion. While not a common material response, the negative Poynting modulus has been observed and investigated in biopolymer networks.^[^ ^6^, ^7^, ^8^ ^]^ The origin of the negative Poynting modulus in biopolymers is rooted in the expulsion of water from their porous networks under deformation allowing them to shrink.^[^ ^10^ ^]^ Similarly, in our metamaterial, the presence of voids allows to circumvent the volume conservation and to stretch the beams under torsion, which leads to negative normal responses. By applying a pre‐compression on our metamaterial the curvatures of the normal response curves in both fixed load and fixed gap experiments (Figure 3a,c) change their sign and magnitude similarly. This indicates that the Poynting response of the structure is independent of whether the gap or the load was fixed, however, its sign and magnitude can be tuned by the level of pre‐compression.

## 5 Programmable Poynting and Shear Moduli

To determine how to program the nonlinear moduli of the metamaterial, we quantify shear and Poynting moduli as a function of pre‐compression. For torsion under fixed gap experiments, the coefficients of the fits to the normal force, *F*_*n*_, and shear force data, *F*_*s*_, in Figure 3c,d, rescaled by *J*/*h* ^2^ and *J*/(*Rh*), give the Poynting (*G*_*n*_) and shear moduli (*G*_*s*_), respectively. For torsion under fixed load experiments, we define the coefficient of the quadratic fits in Figure 3a rescaled by *J*/(*A*_*s*_ *Y*_*eff*_ *h* ^2^) as the Poynting modulus. Figure 3e,f show the Poynting and shear moduli rescaled by Young's modulus of the elastomer as a function of compressive strain for both loading scenarios.

The Poynting modulus in the pre‐buckling regime is negative. For the intermediate pre‐compressions (buckling regime), the Poynting modulus becomes zero. However, by approaching the self‐contact regime, it rapidly increases and reaches a maximum value at δ ≈ δ_*c*_, where the transition from the buckling to the self‐contact regime occurs. The obtained moduli from both loading scenarios coincide as expected, apart from a deviation occurring at this transient deformation. By further increasing the strain *G*_*n*_ decays sharply and approaches zero. Both shear moduli calculated from the two loading scenarios coincide as well (Figure 3f). The shear modulus, *G*_*s*_, first decreases to zero after the transition from the pre‐buckling to the buckling regime. Subsequently, *G*_*s*_ increases sharply at the onset of the self‐contact regime and keeps increasing at higher pre‐compressions but with a lower rate. The absolute value of *G*_*n*_/*G*_*s*_ varies from 0 to about 13 for our metamaterial, whereas for a bulk cylindrical shell with the same dimensions and made of the same elastomer *G*_*n*_/*G*_*s*_ ≈ 0.5, in agreement with the theoretical prediction of *G*_*n*_/*G*_*s*_ = 5/8, for an incompressible isotropic rubber.^[^ ^5^ ^]^ Misra et al. have found that when a rectangular pantographic plate is being twisted around its long axis its normal and torque responses, can be tuned by modifying the geometry of the ligaments and structural parameters of the unit cell [12]. In contrast, we program the Poynting effect and the torque response of our designed structure based on an interplay between buckling of the ligaments and self‐contact between them (Figure 3a– d). Both systems at small torsional angles show linear and quadratic behavior for the shear and normal responses, respectively. The pantographic plates for specific combinations of geometrical parameters can show a transition to negative normal force under torsion. However, in large torsional angles, the responses of the two systems are significantly different. The response of pantographic plates under compression is not studied while for the cylindrical meta‐structure under compression the inverted Poynting effect is observed.

Thus, by tuning the level of pre‐compression we can program the magnitude and sign of the Poynting modulus and even eliminate it, In addition, our structure shows a strong potential for tuning the shear modulus over a wide range.

## 6 Oscillatory Poynting Modulus in Large Deformation

**Figure 4:**
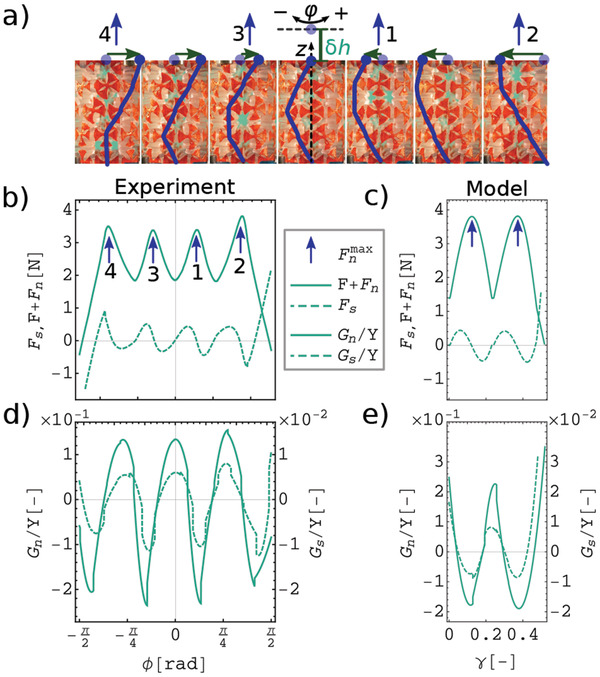
Periodic Poynting moduli in large strain deformation under the pre‐compression of δ = 0.13 in the self‐contact regime. a) Sequence of images showing the internal deformations of the cylindrical structure during one cycle of a large amplitude torsion experiment. b,c) Normal response, *F* + *F*_*n*_, (solid) and shear force, *F*_*s*_, (dashed) as a function of torsion under large torsional deformations, for experiment (b) and model (c). d,e) Rescaled Poynting, *G*_*n*_/*Y*, (solid) and shear, *G*_*s*_/*Y*, (dashed) moduli as a function of torsion for experiment (d) and model(e). (c) and (e) are predictions of the model only for positive shear deformations (γ ⩾ 0) with a fixed gap boundary. The results of negative shear are mirror images of these curves, with four peaks in one deformation cycle.

In Figure 3a– d, we observe deviations from the quadratic response with a nonmonotonic behavior at large torsions. To understand this behavior at large deformations, we follow the structural changes and mechanical responses under large torsional deformation at a fixed pre‐compression in the self‐contact regime (δ_*c*_). **Figure**  **4**a shows sequential images of the structural changes in our experiment. In Figure 4b, we observe a periodic oscillation in both normal (solid line) and shear (dashed line) forces as a function of torsion amplitude. In the course of torsion, one layer slides over another layer by snapping its beams from tilted to vertical and again tilted configurations, causing a local maximum in the normal force. Since the rearrangement occurs layer by layer, the number of the peaks of the normal response is set by the number of layers (*l* = 4). For large deformation experiments we determine the Poynting and shear moduli as *G*_*n*_ = (*h* ^2^/(2*J*))(∂^2^ *F*_*n*_/∂ϕ^2^), and *G*_*s*_ = (*Rh*/*J*)(∂*F*_*s*_/∂ϕ). Figure 4d shows both rescaled *G*_*n*_ (solid line, left axis) and *G*_*s*_ (dashed line, right axis) oscillate between positive and negative values. Negative values of *G*_*s*_ accompanied by negative slopes in the curves of shear force indicating the occurrence of the snap instability (see Video S4, Supporting Information). Thus, snap instability and self‐contact under large torsional deformation result in oscillatory nonlinear moduli that are not allowed in conventional materials and rare in metamaterials.

## 7 Theoretical Predictions versus Experimental Results

We model the experimental system by using a 2D square network of Hookean springs and energy minimization to predict the configuration and mechanical responses of the system. Details of this model are explained in the modeling procedure section. The results predicted by the model successfully reproduce all the qualitative features of the experiments, as shown in the insets of Figures 4d, 2e, 3e, 3f, 4c, and 4e, with minor quantitative differences. For example, the model's maximum values of *G*_*n*_ and *G*_*s*_ are higher than observed experimentally (Figure 3e,f). This can be attributed to neglection of the thickness of the beams' middle point in the model, which excludes the bending possibility at the middle of beams and leads to a higher *G*_*n*_ and *G*_*s*_. Using this model, the onset of the self‐contact regime can be predicted analytically by $\delta_{c}\approx \left(1-\cos\left(\frac{\pi }{2}-2\alpha \right)\right)+\delta_{b}\approx 0.13$, in a good agreement with the experimental observations. Moreover, we analytically predict the square root coupling of compression‐torsion observed in the inverted Poynting experiment as $\gamma =\sqrt{2(\delta -\delta_{b})}$ (Figure 4e, inset). Since shear deformation represents itself as a torsion on the cylindrical shell, by converting shear strain to torsion using ϕ = (*h*_0_/*R*)γ, we predict a coefficient of 5.7 for the square root relation close to the experimental value of 4.9 (Figure 4e). The simple linear spring model identifies buckling and self‐contact as the minimum ingredients to achieve programmable/inverted Poynting effects and confirms the structural origin of the nonlinear responses.

In conclusion, we have engineered a cylindrical metamaterial with programmable Poynting and shear responses. We showed that our designed structure is capable of exhibiting the inverted Poynting effect by translating an axial compression to a nonlinear torsion, in contrast to conventional elastic materials. We also succeeded in programming the Poynting modulus by varying the level of pre‐compression/loading prior to torsion. We switched the sign of the Poynting modulus and tuned its value over a wide range, including even eliminating it. Furthermore, we successfully modeled and studied the system using an energy minimization method. The model identifies buckling of the ligaments and self‐contact as the essential design elements to achieve programmable and inverted Poynting in a metamaterial. Our analytical approach opens avenues for bottom‐up programming of the shear and normal mechanical responses of metamaterials based on self‐contact as a mechanical feedback mechanism. Besides the fundamental importance of understanding nonlinear shear and normal moduli, their programmability provides a groundwork to numerous possible applications in solid mechanics. For instance, our system is capable of translating a unidirectional motion into torsion and of switching the mechanical forces. As these are relevant features of machines, we anticipate applications in designing machine materials, robot hands, mechanical force switches, and rotational actuators with simpler and more efficient mechanism compared to the conventional mechanisms. Additionally, the ability to program the Poynting effect inspires applications in biomechanics where couplings of torsional and axial deformations are ubiquitous (e.g. tendons, cartilages, and cardiovascular systems)^[^ ^21^, ^29^, ^30^, ^31^ ^]^ and may lead to engineer novel biological tissues, implants, and external prosthetic devices functioning under torsion and compression.^[^ ^23^, ^24^, ^25^ ^]^

## 8 Experimental Details and Modeling

### 8.1 Experimental Procedure

**Figure 5:**
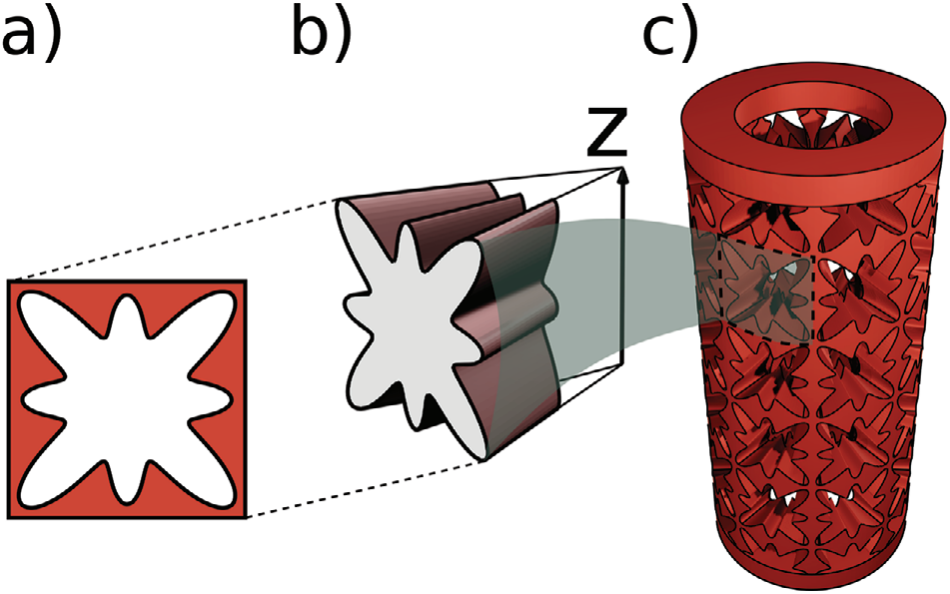
a) The pore contour. b) CAD model of the void, created by extruding the pore contour in radial direction toward *z* axis. c) An isometric view of the CAD model of the cylindrical metamaterial.

The cylindrical beam network is created by extracting an evenly distributed circular pattern of eight voids, with a fourfold symmetry contour, in *l* number of layers from a cylindrical shell with an outer radius of *R*_*max*_ = 12.5 mm, and an inner radius of *R*_*min*_ = 7.5 mm. We use the polar function *s*(*u*) = *c*[(1 − (*a* + *b*)) + *a*cos (4*u*) + *b*cos (8*u*)] to create shape of the pores with a 4‐fold symmetry contour, where, *s*(*u*) is the radius at the polar angle *u*, *x* = *s*(*u*)cos (*u*), and *y* = *s*(*u*)sin (*u*). In this equation, *a* and *b* are the shape tuning parameters, and *c* sets the pore's size. Considering *a* = *b* = 0, we can create a circle with a radius of *c*. Overvelde et al. showed that a 2D network with the flowing parameters for the pore shape of the unit‐cell, *a* = −0.21 and *b* = 0.28 (**Figure** **5**a), exhibits side‐buckling under compression.^[^ ^27^ ^]^ This shape also provides a nonuniform profile for the cross‐section of the beams that form the structure. We use the same parameters to create our unit‐cells. First, in CAD software (Blender 2.8), we place the pore contour (Figure 5a) at the outer surface of our cylindrical shell so that the plane of the pore is parallel to the axes of the shell. Then, we extrude this pore shape in the radial direction towards the center of the cylinder and create the void volume (Figure 5b). Carving a radial array of 8 voids evenly distributed around 2π*rad*, in *l* layers leaves us with a cylindrical network of nonuniform beams composed of 8 × *l* unit‐cells. We set the pore size parameter as *c* = 4.7 mm, which gives the minimum beam thickness of 0.8 mm at the shell's outer surface. We create extra half layers on top and bottom and, finally, clamp the structure with two O‐rings with the same maximum and minimum radii as the shell and a height of 3 mm (Figure 5c). These features enable us to clamp two sides of the cylindrical metamaterial during the measurements. We 3D print the designed cylindrical metamaterial using a Formlab Form2 3D printer and elastic resin v1, with a resolution of 0.1 mm. We 3D print a bulk cylindrical shell with the same elastic resin and dimensions as our cylindrical metamaterial, and perform a compression test to determine Young's modulus of the bulk as *Y* = 3.63 MPa.

### 8.2 Statistical Analysis

We conduct the compression and torsion experiments on our cylindrical metamaterial via an Anton Paar MCR302 rheometer with accuracies of 1μm (longitudinal displacements), 0.05μ rad (angular displacements), 0.005N (normal forces), and 1 nNm (torques). Using a custom made plate‐plate geometry, we clamp the upper and bottom sides of the shell (Figure 5a). The deformation angle, ϕ, is positive when the torsion is clockwise.In this article, we present the data from single tests. Repeating the experiments with the same structures may lead to negligible changes, smaller than 0.1N in force responses and 0.2% in strain rates, which can root in the durability of the elastomer. The analysis on data and visualizations has been performed using Mathematica 11.

### 8.3 Modeling

**Figure 6:**
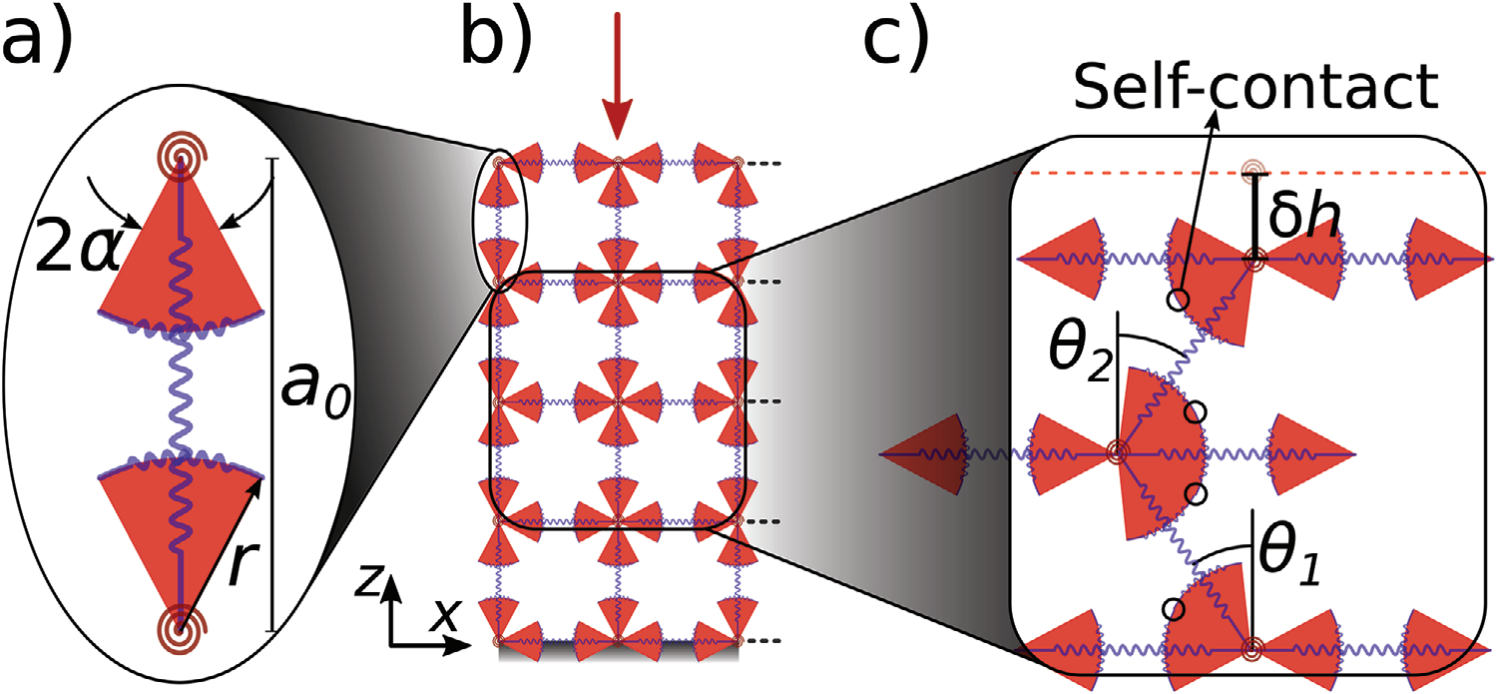
a) Schematics of a non‐uniform beam with associated parameters in our model. b) Schematic of the network of the beams in 4 layers. c) Under the pre‐compression, beams are tilted by θ_*i*_ and when $\theta_{i}\geq \frac{\pi }{2}-2\alpha$ self‐contact occurs.

We model the cylindrical metamaterial as a 2D square network of Hookean elastic beams with the length of *a*_0_ that could either stretch or contract. The beams can bend at the connecting nods. Each beam has a pair of arc‐shaped elastic arms, which are placed at the distance *r* from each end of the beam and symmetrically spread by ±α (**Figure** **6**a,b). We study this model system in a 2‐step deformation procedure: first, compression along the vertical axis, *z* (Figure 6c), and then shear along the horizontal axis (*x*). The connecting arms to the beams are designed to mimic the beam profile's nonuniformity, and they can deliver self‐contact under deformation. Self‐contacts lead to elastic contractions of the arms that produce the reaction forces.

The total elastic energy due to deformation of each beam is composed of stretching energy, $E_{s}=\frac{1}{2}ka_{0}^{2}e^{2}$, and bending energy, *E*_*b*_ = *k*_*b*_θ^2^. To calculate the contribution of self‐contact in the elastic energy we assume that the arc‐shaped arms have the same Hookean coefficient as the straight parts of the beam, *k*, and when subjected to a self‐contact, their curvature remains constant but their length, *s*, changes through change of the arc angle, 2α; thus, δ*s* = 2*r*δα. Since the deflection of the beam is divided between to contacting arcs, we can write δα = δθ/2, where δθ = θ − θ_*c*_ is the deflection of the beam after self‐contact at $\theta_{c}=\frac{\pi }{2}-2\alpha$. Due to such deformation, the energy of each arm changes by $E_{c}=\frac{1}{2}kr^{2}\delta \theta^{2}=\frac{1}{2}k_{c}\delta \theta^{2}$, which gives the elastic coefficient of the self‐contact as *k*_*c*_ = *kr* ^2^. So the total energy for *l* layers of the vertical beams is:

(1) $$\begin{array}{c} E=k_{b}\sum_{i=1}^{l}\theta_{i}^{2}+\frac{1}{2}ka_{0}^{2}\sum_{i=1}^{l}e_{i}^{2}+kr^{2}\sum_{i=1}^{l}\delta \theta_{i}^{2}H\left[\delta \theta_{i}\right] \end{array}$$

Since bending and self‐contact occur symmetrically at both nodes of each vertical beam, their energy terms do not have the pre‐factor 1/2. Heaviside step function, *H*, represents the self‐contact interactions; *H*[*x*] is 0 for *x* < 0 and 1 for *x* ⩾ 0.

By considering a periodic boundary condition in the horizontal direction, we can model a closed structure such as a cylinder. Since each horizontal beam is joined with two beams at its right and left sides, alteration of its orientation is not favorable. Under this condition and since we apply the compression and shear deformations uniformly at the top layer, horizontal beams remain horizontal. Thus we can infer one column structure's solution to the whole system and define the total energy of a system with *n* unit‐cells in each layer is *E*_*tot*_ = *nE*.

We use *n* = 8, *l* = 4, α= 0.54 rad, and *r* = 4.7 mm, mimicking our experimental structure. The spring constant of the whole cylinder, *K*, is given by *K* = *Y*_*eff*_ *A*_*s*_/*h*_0_. Since the cylinder consists of l layers with 8 beams in each layer, the spring constant for each beam can be calculated by *k* = *Kl*/8. We estimate the spring constant from the force response in the compression experiment before buckling as *k* = 1331 Nm^−1^. To estimate the bending stiffness of a beam with a rectangular cross‐section, we can use use *k*_*b*_ = *M*/θ = *YI*/(*r*_*c*_θ) ≈ (2*YI*/*l*_*b*_), where *I* is the moment of inertia, *l*_*b*_ is the length of the beam, and *r*_*c*_ is its bending curvature. By assuming that the beam has the same thickness and width as at the thickness and width of the hinge part, we can obtain the moment of inertia. This estimation predicts an order of magnitude of 10^−4^Nm for the beams' bending stiffness. We finally calibrate the bending stiffness at *k*_*b*_ = 8.4 × 10^−4^Nm to obtain the buckling instability at the same compression strain as in the experiment (δ_*b*_ = 0.012). We obtain the modeled system's configuration by numerically minimizing its energy (Equation (1)) under different boundary conditions. Numerical minimizations are performed by Mathematica 11 using the Nelder–Mead method and a working precision of 15 digit numbers. We use the presented model to mimic the torsion‐free compression, clamped compression, and fixed gap torsion experiments. In our spring model, we can tune the onset of buckling and self‐contact transitions by changing the stiffness coefficients and geometrical properties of beams, such as α. For instance, increasing the bending stiffness of the beams, while keeping the stretching stiffness constant, leads to an increase in the onset of the buckling regime. Additionally, by increasing α the width of the beam profile increases and therefore, transition to the self‐contact regime occurs at a lower pre‐compression strain. These parameters would provide a high potential for tuning the mechanical responses of the structure.

To use our theoretical model in a continuum limit, we need to introduce a higher‐order interaction term due to mechanical interaction between different layers. This second‐order terms can be added to the energy function in the form of (θ_*i*_ − θ_*i* + 1_)^2^. Using such an energy function at the continuum limit gives rise to second gradient terms in the equation of motion, which can describe the second gradient effects in the system. A similar approach has been used to describe extensible beams^[^ ^32^ ^]^ and the pantographic plates under different modes of deformations, such as longitudinal extensions of the plate, in‐plane shearing, and extension combined with the in‐plane rotation of an edge.^[^ ^32^, ^33^ ^]^

## Conflict of Interest

The authors declare no conflict of interest.

## Supporting information

Supporting Information

## Data Availability

The data that support the findings of this study are available from the corresponding author upon reasonable request.
