# Supplementary material for: Inverted and Programmable Poynting Effects in Metamaterials
Source: Adv Sci (Weinh). 2021 Aug 17;8(20):2102279. doi: 10.1002/advs.202102279 (PMC8529495; doi:10.1002/advs.202102279)
Supplement: Supplementary file 1 — Supporting Information [file ADVS-8-2102279-s001.pdf]

© 2021 Wiley-VCH GmbH

ADVANCED SCIENCE
Open Access

Supporting Information

for *Adv. Sci.,* DOI: 10.1002/advs.202102279

Inverted and Programmable Poynting Effects in Metamaterials

*Aref Ghorbani, David Dykstra, Corentin Coulais, Daniel Bonn, Erik van der Linden, and Mehdi Habibi**

Click here to access/download
Supporting Information
S1_Video_Introduction_v0(Compressed).avi

Click here to access/download
Supporting Information
S2_Video_Compression_Presentation(Compressed).avi

Click here to access/download
Supporting Information
S3_Video_ProgramablePoynting(Compressed).avi

Click here to access/download
Supporting Information
S4_Video_OscillatoryPoynting(120MB)(Compressed).avi
